# Analytical Quality by Design (AQbD) Approach to the Development of In Vitro Release Test for Topical Hydrogel

**DOI:** 10.3390/pharmaceutics14040707

**Published:** 2022-03-26

**Authors:** Réka Szoleczky, Mária Budai-Szűcs, Erzsébet Csányi, Szilvia Berkó, Péter Tonka-Nagy, Ildikó Csóka, Anita Kovács

**Affiliations:** 1 Egis Pharmaceuticals Plc., Laboratory of Finished Product Analytical Development 3, Bökényföldi Str. 116-120, 1165 Budapest, Hungary; szoleczky.reka@egis.hu (R.S.); tonka.nagy.peter.gabor@egis.hu (P.T.-N.); 2 Institute of Pharmaceutical Technology and Regulatory Affairs, University of Szeged, Eötvös Str. 6, 6720 Szeged, Hungary; budai-szucs.maria@szte.hu (M.B.-S.); sooscsanyi@gmail.com (E.C.); berko.szilvia@szte.hu (S.B.); csoka.ildiko@szte.hu (I.C.)

**Keywords:** analytical quality by design, in vitro release test, USP apparatus IV with semi-solid adapter, topical gel, diclofenac sodium

## Abstract

The aim of our study was to adapt the analytical quality by design (AQbD) approach to design an effective in vitro release test method using USP apparatus IV with a semi-solid adapter (SSA) for diclofenac sodium hydrogel. The analytical target profile (ATP) of the in vitro release test and ultra-high-performance liquid chromatography were defined; the critical method attributes (CMAs) (min. 70% of the drug should be released during the test, six time points should be obtained in the linear portion of the drug release profile, and the relative standard deviation of the released drug should not be over 10%) were selected. An initial risk assessment was carried out, in which the CMAs (ionic strength, the pH of the media, membrane type, the rate of flow, the volume of the SSA (sample amount), the individual flow rate of cells, drug concentration %, and the composition of the product) were identified. With the results, it was possible to determine the high-risk parameters of the in vitro drug release studies performed with the USP apparatus IV with SSA, which were the pH of the medium and the sample weight of the product. Focusing on these parameters, we developed a test protocol for our hydrogel system.

## 1. Introduction

Topical dermal preparations can be used to achieve both local and systemic effects. For the former, the effect may be superficial, but sometimes deeper tissue penetration may be required. “The diversity of topical products is very wide given the complex nature of skin, the range of conditions to be treated and the variety of patients and their needs” [1] (p. 5). Topical products can be: creams, ointments, gels, pastes, poultices [2], suspensions, lotions, foams, sprays, aerosols, solutions, and transdermal delivery systems (TDS) [3].

The rate of drug release from the topical product and its retention are key factors in the development of the effect. In the case of a non-topical target, the development of the effect is influenced by the physicochemical properties of the active pharmaceutical ingredient (API) and the effect of the formulation on penetration as well.

### 1.1. Product Quality Tests for Topical and Transdermal Drug Product

The concept of critical quality attributes (CQAs) was defined in the ICH Q8 [4]; these are the physical, chemical, or microbiological properties that can be measured in order to ensure the desired product quality (parameters should be within an appropriate limit, range, or distribution). These CQAs may include drug release, preservative type and content, purity, pH, rheological properties, etc. [5].

**Figure 1:**
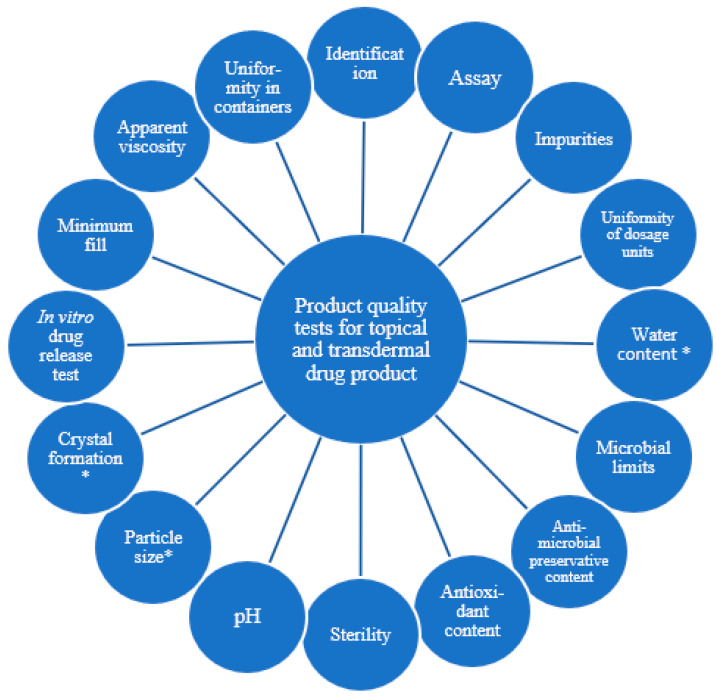
Product quality tests for topical and transdermal drug products. * This test is generally formulation dependent.

The Pharmacopeial Forum [6] and USP monograph [3] described the product quality test recommendations for topical, dermal, and transdermal drug products (Figure 1). The factors mentioned in this description can serve as cornerstones for development in order to ensure the optimal quality of the product, especially in the case of generic drug development.

The in vitro release test is a well-known technique for analyzing a semi-solid dosage form. With this measurement, the semi-solid dosage form is placed on the upper side of an artificial, inert membrane in the donor chamber in contact with a medium in a reservoir/cell, and the API diffuses through the formulation, across the membrane, into the reservoir. After that, the drug release rate (released per unit area (µg/cm^2^) against the square root of time, yielding a straight line), the cumulative amount of API released at the last sampling time of the linear portion, and lag time parameters should be determined [1,7,8].

During development, an in vitro dissolution/release test can be used to determine the formulation factors that may influence the bioavailability of the API. In addition, at the beginning of generic development, we can use the in vitro dissolution/release test as a reverse engineering tool to copy the RLD (reference listed drug), and in this way it can be a critical tool for highlighting the differences between the generic product and the RLD. In order to perform the BCS-based (biopharmaceutics classification system) biowaiver study, we need to determine and compare the in vitro dissolution/release performance of the RLD and the generic product. After the manufacturing process, when the composition of the generic product has already been defined, and if the authorities have approved it, the in vitro dissolution methods can be used to provide batch-to-batch quality measurements [9].

International recommendations for the in vitro release process for a semi-solid dosage form are also described in the USP <1724> Semi-Solid Drug Products—Performance Tests [8], <724> Drug Release [10], and in the Japanese Pharmacopoeia 6.10 Dissolution Test [11].

The SUPAC-SS (scale-up and post-approval changes) guidance [7] focuses on post-approval changes in excipients in the drug product after administration and ensures product sameness and quality, describing the details of the in vitro release comparison test. For the comparison of the pre-change lot (P) and the post-change lot (T), measurements should be made by two IVRT (in vitro release test) runs performed on two different days: in the first run, three cells of P and three cells of T are included, while the second run is the same as the first run, but with an opposite arrangement of the P and T samples on the diffusion cells. After the IVRRs (in vitro release rates) of P and T were evaluated and divided (T/P ratio), the confidence interval (90%) was calculated to order these 36 individual T/P ratios from lowest to highest. Thereafter, the eighth and twenty-ninth ordered individual ratios should fall within the limits of 75% to 133.33% [7].

### 1.2. Quality by Design Usage in the Development of Topical Semi-Solid Dosages

The quality by design approach was defined in the ICH (International Council for Harmonisation of Technical Requirements for Pharmaceuticals for Human Use) Q8 (R2) guideline as “a systematic approach to development that begins with predefined objectives and emphasizes product and process understanding and process control, based on sound science and quality risk management” [4]. Quality needs to be designed into the product, not “tested into the product”. Using this risk-based approach during pharmaceutical development, the outcome will be a well-known product and process. As a first step, the quality target product profile (QTPP) and the critical quality attributes (CQAs) have to be defined on the basis of prior knowledge. Afterwards, the critical material attributes (CMAs) and critical process parameters (CPPs) have to be determined by carrying out the risk assessment methods mentioned in the ICH guideline Q9, which are linked to the CQAs of the drug product. This Q9 guideline mentioned a couple of risk management methods and tools, including the Ishikawa diagram, Pareto analysis, failure mode effects analysis (FMEA), and design of experiments (DoE) methods [4,5,12,13,14,15,16].

The analytical quality by design (AQbD) concept, an extension of quality by design (QbD) [17], results in a well-understood, fit-for-purpose, and robust method that consistently delivers the intended performance throughout its lifecycle [13,18,19,20]. 

During the AQbD method development, the gained and reliable knowledge provides adequate evidence to meet the performance requirements, such as the selection of the critical parameters or the method validation parameters, and enhanced understanding of the product control strategy precludes unnecessary tests. The application of AQbD can be used to support post-approval changes to analytical procedures through activities such as science- and risk-based change management.

The goal of our present study is to adapt the AQbD approach to design an effective IVRT method development using USP apparatus IV (flow-through cell with a semi-solid adapter) [8] for a topical hydrogel.

## 2. Materials and Methods

### 2.1. Materials 

Diclofenac sodium salt was purchased from Molar Chemicals Ltd. (Halásztelek, Hungary). Propylene glycol and hypromellose (HPMC) were provided by Hungaropharma Ltd. (Budapest, Hungary).

The water used was filtered and deionized using the ELGA PURELAB Chorus 1 lab water purification system (ELGA LabWater Headquarters, Lane End, United Kingdom). Di-sodium hydrogen phosphate dihydrate, sodium hydroxide, and sodium chloride were provided by Molar Chemicals Ltd. (Halásztelek, Hungary). Potassium dihydrogen phosphate was obtained from Thomasker (Budapest, Hungary). All the other chemicals were of analytical grade or equivalent. The dissolution media for the IVRT test were pH 7.4 PBS (composition: 0.007 M Na_2_HPO_4_ × 2 H_2_O, 0.001 M KH_2_PO_4_, 0.137 M NaCl, adjusted to pH 7.4 ± 0.05 with cc. H_3_PO_4_), pH 6.9 PBS (composition: 0.007 M Na_2_HPO_4_ × 2 H_2_O, 0.001 M KH_2_PO_4_, 0.137 M NaCl, adjusted to pH 6.9 ± 0.05 with cc. H_3_PO_4_), pH 7.9 PBS (composition: 0.007 M Na_2_HPO_4_ × 2 H_2_O, 0.001 M KH_2_PO_4_, 0.137 M NaCl, adjusted to pH 7.9 ± 0.05 with 1 M NaOH), pH 7.4 PBS + NaCl (0.007 M Na_2_HPO_4_ × 2 H_2_O, 0.001 M KH_2_PO_4_, 0.411 M NaCl, adjusted to pH 7.4 ± 0.05 with cc. H_3_PO_4_), and pH 7.4 PBS–NaCl (0.007 M Na_2_HPO_4_ × 2 H_2_O, 0.001 M KH_2_PO_4_, 0.046 M NaCl, adjusted to pH 7.4 ± 0.05 with cc. H_3_PO_4_).

Acetonitrile (HPLC gradient grade) was acquired from Merck (Darmstadt, Germany). Methanol (HPLC gradient grade) was purchased from Honeywell International Inc. (Charlotte, NC, USA).

### 2.2. Methods

During our work, different quality management tools were used (Ishikawa diagram and failure mode and effects analysis) in order to have a full scope of applied analytical methods (with the IVRT and UHPLC methods). For all the statistical analysis of variance and the design of experiments, the Statistica 13 software (Copyright 1984–2018 TIBCO Software Inc., Paolo Alto, CA, USA) was used.

#### 2.2.1. USP Apparatus IV: Flow-Through Cell with a Semi-Solid Adapter

A semi-solid adapter or insertion cell (diffusional surface area: 1.54 cm^2^) was used with USP apparatus IV (Sotax CE7 smart with CY 7 piston pump, Sotax Corporation, Westborough, MA, USA) to model the in vitro drug release from diclofenac sodium topical hydrogel. The donor compartments of the semi-solid adapters (available in different sizes: 400 µL, 800 µL, and 1200 µL) were filled with the topical product, and afterwards the Teknokroma cellulose membranes (pore size of 0.45 µm) (previously soaked in pH 7.4 PBS for 30 min) were fitted into the screw constraint and were placed over the surface of the sample compartments. The adapters with the membrane facing down were loaded into the 22.6 mm tablet cells prefilled with glass beads (1 mm glass beads) [21,22]. The apparatus IV system was used in an “open loop” configuration. The medium was deaerated pH 7.4 PBS, and the flow rate was 4 mL/min or 8 mL/min. The test temperature was 32 ± 0.5 °C and samples were collected (Sotax C 615 fraction collector, Sotax Corporation, Westborough, MA, USA) at 0.5, 1, 2, 3, 4, 5, and 6 h. We used 400 µL and 1200 µL semi-solid adapters for the IVRT development. Samples were analyzed using UHPLC (ultra-high-performance liquid chromatography).

The drug release rates were calculated using USP general chapter 1724 [8].

#### 2.2.2. Ultra-High-Performance Liquid Chromatography Analysis

The concentration of diclofenac sodium was determined using a Waters Acquity I-Class UHPLC system with a photo diode array (PDA) detector set to the wavelength of 240 nm. Chromatographic separation was performed using an Acquity UPLC BEH UHPLC column (2.1 mm × 50 mm, 1.7 µm, 130 Å, Waters Corporation, Milford, MA, USA), the temperature was maintained at 40 °C, and the mobile phase was a mixture of methanol and potassium dihydrogen phosphate buffer (pH 2.5; 20 mM) (36/64 *v/v*). The potassium dihydrogen phosphate buffer was filtered through a 0.22 μm filter. The degassing of the mobile phase was achieved through the ultrasonication of the eluent for up to 5 min. The run time was set to 3 min. The flow rate was 0.45 mL/min, and the injection volume was 2 μL. For each in vitro release study, calibration was established in the concentration range of 4 to 100 μg/mL (R² ≥ 0.995). The chromatographs were analyzed using Empower 3 (copyright 2010 Waters Corporation).

#### 2.2.3. Analytical Quality by Design

A general workflow can be traced for the implementation of AQbD: first, the definition of the analytical target profile (ATP) and critical analytical attributes (CAAs); after these, the identification of the critical method parameters (CMPs) (for example, Ishikawa diagram) and a risk assessment analysis (for example, FMEA) should be performed, followed by a design of experiments (DoE). Finally, through a response surface analysis, the establishment of the design space pertaining to the method is also referred to the method operable design region (MODR) [23,24,25,26,27,28]. 

#### 2.2.4. Definition of the Analytical Target Profile

“The ATP states the required quality of the results produced by a procedure in terms of the acceptable error in the measurement” [29]. Therefore, the first step in the AQbD-based analytical development is to define the ATP, which is analogous to QTPP. It should be established before selecting the technology and starting the development of the method, and its intended purpose should be defined [17,24,26].

The ATP includes the product to be tested (API name, dosage form, API content, the definition of the route of administration, matrix, etc.), the range of analyte concentration, the allowable error for the measurement, the allowable risk of the criteria not being met, and the confidence that the measurement uncertainty and risk criteria are met. “The ATP criteria are independent of the technique, allowing an analyst to select any technique that is capable of providing the performance needed to meet the ATP criteria” [29]. Diclofenac sodium, a non-steroidal anti-inflammatory drug [30], was used as a model drug in the hydrogel system to adapt the AQbD approach.

#### 2.2.5. Definition of the Critical Method Attributes and Critical Method Parameters

The second step in the AQbD-based development is to determine CMAs. On the basis of our prior method development knowledge and data, CMAs are derived from the ATP. CMAs are the elements of method performance which need to be measured and/or evaluated to ensure that the desired data will be provided. CMAs are analogous to CQAs in drug development [31]. 

After the definition of CMAs, all the method parameters can be summarized systematically with the help of an Ishikawa diagram [16]. The Ishikawa diagram, also called a fishbone diagram, is the most adopted technique for the risk analysis of cause–effect phenomena. The aim of this method is to summarize all influencing factors during a brainstorming session, and then to categorize and to visually represent MPs.

#### 2.2.6. Establishing Failure Mode Effects Analysis (FMEA)

Failure mode effects analysis is an important risk assessment method defined in the ICH Q9 guideline, which states that it “provides for an evaluation of potential failure modes for processes and their likely effect on outcomes and/or product performance” [16]. In the risk matrix, we can estimate the effect and the risk of the method parameters with regard to the method performance.

The outcomes of an FMEA are the risk priority numbers (RPNs). They can be used to rank risks from the FMEA analysis. RPNs are calculated by multiplying occurrence (O), severity (S), and detection (D) indexes. O is the occurrence of failure or the likelihood of an event occurring. S is the severity scale that could be based on the impact that the sources of variability have on the analytical procedure measurement (ability to meet the ATP criteria). D is detectability or the ease with which a failure mode can be detected [15,32,33]. Table 1 describes the rankings of severity, occurrence, and detectability of effect.

On the basis of the RPNs, the following classes of risks can be distinguished [32]:

- Low (acceptable) 1 ≤ RPN ≤ 29;
- Medium (to be considered) 30 ≤ RPN ≤ 59;
- High (not acceptable) 60 ≤ RPN ≤ 125.

The method parameters which were classified as medium or high risk in the FMEA should be considered to be critical method parameters (CMPs).

#### 2.2.7. Design of Experiments (DoE) for IVRT Method

The DoE method is a modelling tool for assessing possible interactions between the factors influencing the drug development process and, thus, the quality of the final product. CMPs must be chosen as independent variables and CMAs as dependent variables in the factorial design process.

After the FMEA and the preliminary experiments, a 2^3^ full factorial design was performed for the optimization of an IVRT method for a diclofenac sodium hydrogel formulation. A first-order polynomial model (Equation (1)) was generated in order to investigate the linear response surface, which can describe the principal effects and interactions between the identified variables.

(1) $$y=a_{0}+a_{1}x_{1}+a_{2}x_{2}+a_{3}x_{3}+a_{12}x_{1}x_{2}+a_{23}x_{2}x_{3}+a_{13}x_{1}x_{3}$$

where a_0_ is the intercept, a_1,2,3_ are the regression coefficients values, and x_1_, x_2_, and x_3_ correspond to the independent factors.

#### 2.2.8. Determination of the Osmolality of Different Media

The analysis was carried out with Knauer semi-micro automatic osmometer, digital/L model (osmolality range: 0–2000 mOsmol/kg) using the freezing point depression method. Two-point calibration was performed with 0 mOsmo l/kg deionized water (ELGA LabWater Head Quarters, Lane End, United Kingdom) and 400 mOsmol/kg calibration solution (A01241-1, Lot 14384042, Knauer). To determine the osmolality of the 150 µL media, each sample was analyzed twice.

#### 2.2.9. Performing Membrane Inertness Test

The membrane used during the IVRT measurements should not absorb the API, should be compatible with the receptor media, and should not be an obstacle to drug diffusion. The measurements were carried out with Teknokroma ME Cellulose 0.45 µm and Millipore PES membrane 0.45 µm with three parallels. Here, 150 mL of pH 7.4 ± 0.05 PBS solution was incubated at 32 °C, stirred at 100 rpm and spiked with 2 mL of 400 µg/mL diclofenac–sodium stock solution that was dissolved in methanol. After two minutes, samples of 1 mL were taken from the 150 mL of pH 7.4 ± 0.05 PBS solutions. After the samples were taken out, one membrane was immersed in each vessel. As the next step, the vessels were covered and stirred at 100 rpm for six hours at 32 °C. After six hours, the sample-taking procedure was repeated. The drug content of the samples was measured by UHPLC.

#### 2.2.10. Discriminatory Power of the In Vitro Release Test Method

The discriminatory power of the IVRT method is built upon three performance characteristics: sensitivity, specificity, and selectivity. The details of these three concepts are described as follows:

A sensitive IVRT method should be able to discriminate diclofenac sodium release rates from similar formulations. In our work, the IVRT method can be qualified as sensitive if the mean of diclofenac sodium release rate from the 0.5% test hydrogel was lower, and the mean of diclofenac sodium release rate from the 2% test hydrogel was higher than the release rate of the diclofenac sodium gel 1% reference product.

In other respects, the specificity of the IVRT method was shown by plotting the relationship between the three formulation concentrations and the average IVRT release rate. This relationship function should be linear (regression coefficient: R^2^ ≥ 0.90) [1,23,34].

For selectivity testing, we used the statistical approach of Wilcoxon rank sum/Mann–Whitney rank test described in the SUPAC-SS guidance. This is used to calculate the 90% confidence interval for the ratio of the slopes between the test hydrogel (0.5 and 2%) and the reference (1%) batches. To determine the inequivalence between the test and the reference products, those ratios should not lie within the limits of 75–133.33% [7,8,34].

## 3. Results

### 3.1. Definition of ATP and Determination of CMAs

In order to support the development of the formulation on the analytical side, we need adequate analytical methods that should guarantee the quality of the product. Accordingly, the IVRT should be sensitive to the changes and alterations in the formulations, and the analytical measurements must be able to accurately and precisely quantify the API in IVRT samples. Therefore, we defined these targets in the ATP (Table 2).

An UHPLC analytical technique was chosen to measure the IVRT samples because the matrix of the topical gel has a UV active excipient and UHPLC is capable of providing selective, precise, and accurate results to quantify the amount of drug in the release media. UHPLC is a more reliable and widely used technique and it is capable of satisfying the ATP requirements.

CMAs and ATPs may be treated as performance requirements because CMAs represent a link between the purpose of the method and the performance criteria according to the ATP [17]; therefore, CMAs were derived from ATPs. Table 3 demonstrates the potential CMAs affecting the method performances along with a justification for each of them. In summary, the ATP objectives and the CMA requirements of the method were chosen.

CMAs were derived from ATPs, and five key CMAs were determined (Table 3), including (1) minimum 70% of diclofenac sodium should be released from the topical gel within six hours, (2) six time points should be obtained in the linear portion of the drug release profile, (3) the relative standard deviation in the computed released amount of the six vessels must be less than or equal to 10%, (4) the accuracy must be greater than or equal to 98% and less than or equal to 102% at three concentration levels (50, 100, and 200%), and (5) the USP plate count of the column (column efficiency) must be greater than or equal to 3000. 

The first and the second CMAs were chosen on the basis of the draft guideline on the quality and equivalence of topical products. While establishing the ATP, there is a significant—practically inseparable—connection with the QTPP because the first and the second CMAs were formed by the product and the analytical method.

In order to ensure the good reproducibility of the IVRT method, the apparatus of the IVRT should have a relative standard deviation in the computed released amount of the six vessels that is less than or equal to 10%.

Once the ATP has been defined, an analytical technique capable of meeting the ATP requirements should be selected. In this study, we focused on the performance of the in vitro release test method by using USP apparatus IV (with a semi-solid adapter) device because according to the EMA guideline [1], it is easier to meet the “6 time points should be obtained in the linear portion of the drug release profile” [1] criterion.

### 3.2. Identification of the MP using the Ishikawa Diagram

**Figure 2:**
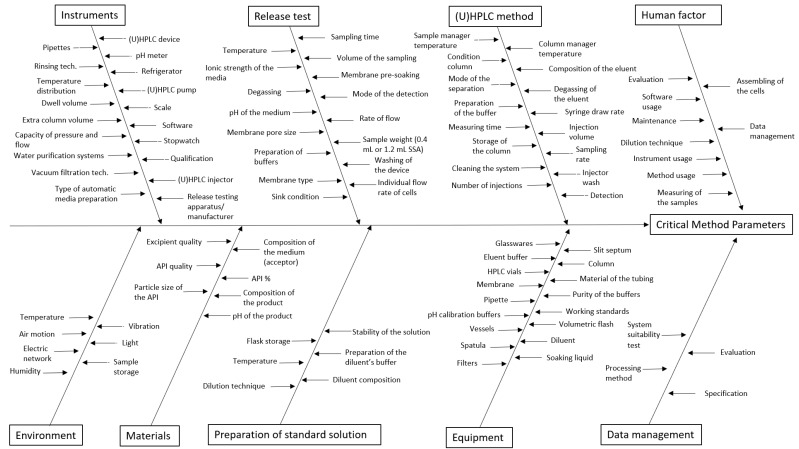
Ishikawa diagram illustrating method parameters that may have an impact on the method attributes.

According to prior knowledge, our next step was to systematically collect all the MPs that could influence a failure concerning the IVRT method. For this, we used the Ishikawa diagram as a risk assessment tool to identify potential variables that could have an impact on CMAs (Figure 2) [15]. With the help of the Ishikawa diagram, more than 100 method parameters were identified that can influence the method performance and the quality of the method’s results.

### 3.3. Initial Risk Assessment using FMEA (Effects of MPs on CMAs)

FMEA was used to establish and prioritize a cause–effect relationship between CMAs and MPs. The fishbone diagram and the FMEA table shown in this article were the results of brainstorming among research pharmacists and analysts. During the FMEA analysis, the possible effects of MPs on CMAs were investigated. The analysis was carried out in the case of all the MPs one by one. The initial risk assessment aims to identify the potential CMPs (that were assigned the highest RPNs), which will be investigated during the preliminary experiments. 

Based on the literature data and our prior method development knowledge, the highest risks (RPN ≥ 60) were identified (see Table 4) using FMEA, including ionic strength (osmolality), the pH of the media, membrane type, rate of flow, sample weight (volume of the SSA), individual flow rate of cells, API% (0.5, 1 and 2%), and the composition of the product. During the screening process, we examined the impact of only the highest scoring parameters on the CMAs independently from each other as a preliminary study.

### 3.4. Carrying out Preliminary Experiments

On the basis of the FMEA and the EMA guide [1] recommendations, in our study, the impact of CMPs, categorized to be high risks, on CMAs was investigated.

#### 3.4.1. In Vitro Release Test Study Design with USP Apparatus IV

A draft guideline on the quality and equivalence of topical products described the IVRT study design [1]. According to this draft guideline, the measurement planning started with choosing the medium and confirming the sink condition. This was followed by the selection of the membrane. The data in Table S1 verify that the sink condition criterion (solubility of the API in pH 7.4 PBS divided by the maximum concentration value of the API in the receptor medium (mg/mL) > 3) is met for USP apparatus IV (3.2 mg/mL). The sink condition in pH 7.4 PBS medium was confirmed according to the literature data [23]; therefore, it was not a CMP.

The results obtained from performing the membrane inertness study (see Section 2.2.9) showed that the Teknokroma ME Cellulose membrane did not act as a rate-limiting barrier to diclofenac sodium diffusion, since the recovery was 100.1 ± 3.7%.

#### 3.4.2. Investigation of the Rate of Flow and Sample Weight with the One-Factor-at-a-Time (OFAT) Method

The one-factor-at-a-time method is the easiest way to examine the impacts of several factors. With this method, it is always only one factor that is changed at a time, and all the other conditions remain the same. The advantage of this method is that the impacts of the factors can be evaluated individually, regardless of one another. The disadvantage of OFAT is the inability to detect interactions between parameters. The importance of the OFAT method is significant at the start of the AQbD (screening), and DoE is recommended for the optimalisation of the method.

**Figure 3:**
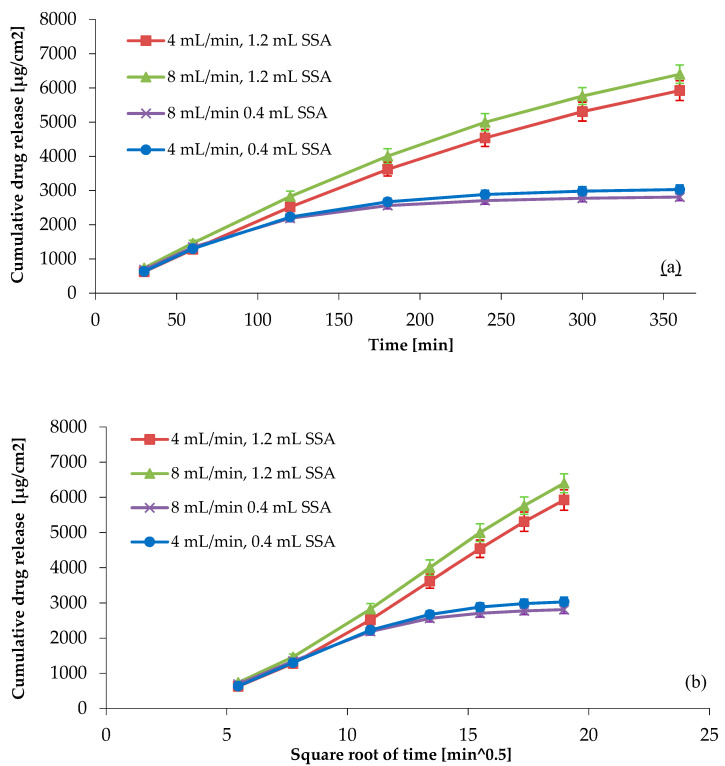
(**a**) Cumulative drug release per unit area in linear time scale, (**b**) cumulative drug release per unit area plotted against square root of time. Instrument: USP apparatus IV.

The effect of the flow rate and the volume of the semi-solid adapter (the weight of the product) can be found in Figure 3. We could observe that the flow rate does not have a significant impact on the release of the API from the diclofenac sodium hydrogel product; however, the volume of the semi-solid adapter does. These results were substantiated by the analysis of variance (*p* < 0.05, main effects analysis of variance (ANOVA), Bonferroni post hoc test). 

The IVRT was carried out with a 1.2 mL semi-solid adapter, and the drug release follows the Higuchi square root law, which is mainly controlled by diffusion. The release from the product only depends on the API’s capacity to diffuse through the membrane. The IVRT carried out with a 0.4 mL semi-solid adapter does not meet the requirements described in the ATP, as instead of six, only three points could be obtained in the linear portion of the drug release profile, but the means of operation of apparatus IV allows us to apply more time points in the linear region in order to meet the criterion “6 time points should be obtained in the linear portion of the drug release profile” [1] without changing the release profile.

#### 3.4.3. Effect of pH and Osmolality on Drug Release from Topical Hydrogel

The diclofenac sodium hydrogel product contains hydroxypropyl methylcellulose (HPMC) as a gelling agent. HPMC is a water-soluble, nonionic, enzyme-resistant cellulose ether. Being nonionic, it allows for pH-independent release if the API itself is not sensitive to pH change [30]. As diclofenac sodium is a derivative of phenylacetic acid (pKa = 4.0), the pH value changes in the medium have a strong effect on its solubility [35]. 

In the case of matrix tablets [36] containing these polymers, ionic strength, and thus the osmolality, was an influencing factor regarding the degree of the release. This phenomenon is explained by the fact that, at a certain point, the increased ion concentration hinders the hydration of the polymer up to a level where forming a continuous gel layer becomes impossible [36].

**Figure 4:**
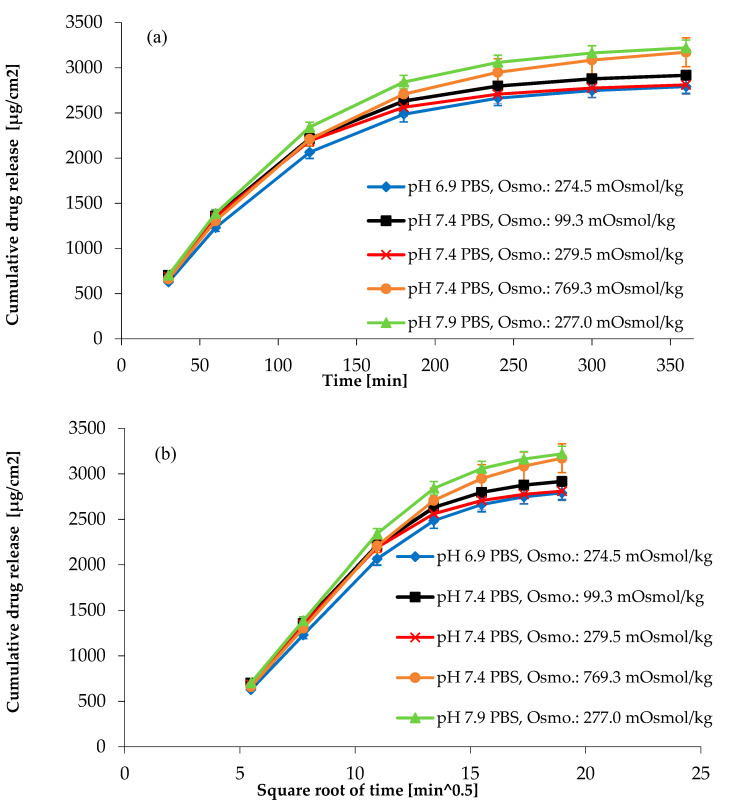
(**a**) Cumulative drug release per unit area in linear time scale, (**b**) cumulative drug release per unit area plotted against square root of time. Instrument: USP apparatus IV.

Due to the reasons mentioned above, the impacts of the osmolality and the pH of the medium on the API release from the gel matrix were categorized as high-risk factors during the risk analysis. The composition of the medium can be found in Section 2.1, Figure 4, and the results of the osmolality test of the medium are shown in Table S2.

Figure 4 and the analysis of variance (Table S3) also show that the impact of pH on API release is significant (*p* = 0.0001, main effects ANOVA, Bonferroni post hoc test), but the effect of the osmolality of the medium is not significant (*p* < 0.05, main effects ANOVA, Bonferroni post hoc test). It can be seen that the RSD% of the computed released amount of the six vessels was less than 10% (Table 5).

### 3.5. The 2^3^ Full Factorial Design for the IVRT Method

On the basis of FMEA and OFAT, the pH of the medium and the sample weight of the product remained high-risk parameters to be examined by an additional modelling method, the design of experiments (DoE). The DoE is a modelling tool for the investigation of a possible interaction between the factors influencing the drug development process and, thus, the quality of the final product. The high-risk CMPs must be chosen as independent variables and CMAs as dependent variables in the factorial design process (Table 6). In the preliminary risk assessment, the flow rate was not a critical parameter, but considering our previous experiences, the sample volume and the flow rate may have a combined effect; therefore, we examined the flow rate as an independent variable in our factorial design.

The flow rate (X1), volume of SSA (X2), and the pH of the medium (X3) were chosen as independent factors and the IVRR (Y1) and release efficiency in 6 h (Y2) were dependent factors. With the preliminary experiments, the flow rate (mL/min) was not found to be a CMP, although, apart from the main effects, two-way or/and three-way interactions can be significant. The DoE was developed by using Statistica 13 software.

From the results of 2^3^ full factorial statistical analysis (*n* = 5 per analysis), it can be seen that the main factors X2 (the volume of the SSA) and X3 (pH) exert a significant effect (*p* < 0.05) on Y1 (IVRR) (Table 7 and Figure S1). The mathematical model shows a good correlation, with R^2^ = 0.96607.

The equation was as follows:

$$Y_{1}=365.9818+2.1168{\;X}_{1}+79.0443{\;X}_{2}+11.9503{\;X}_{3}+3.4333{\;X}_{12}\;-1.7938{\;X}_{13}+0.2698{\;X}_{23}-3.6383{\;X}_{123}$$

On the basis of our results, the combination of the highest pH (7.9) and the highest volume of SSA (1.2 mL) gives us the highest IVRR (µg × cm^−2^ × min^−0.5^) (Figure S2 and Table 7).

Analyzing the effect of the factors on the release efficiency in 6 h, the mathematical model shows a good correlation, with R^2^ = 0.92047. The fitted equation was as follows:

$$Y_{2}=88.2920+0.9240{\;X}_{1}-11.2010{\;X}_{2}-0.7755{\;X}_{3}+0.4840{\;X}_{12}-0.6565{\;X}_{13}\;-0.4535{\;X}_{23}-0.5575{\;X}_{123}$$

In other respects, the statistical analysis shows (Table 8 and Figure S3) that only one main factor X2 (the volume of the SSA) has a significant effect (*p* < 0.05) on release efficiency in 6 h (Y2). The other factors did not have a significant effect on Y2.

It can be also seen that the highest volume of SSA (1.2 mL) gives us the highest release % (Figures S4 and S5).

### 3.6. Updating the FMEA Table

Given these results, the sink condition criterion was not a CMP (3.4.1), and the cellulose membrane did not act as a rate-limiting barrier (3.4.1) to diclofenac sodium diffusion, since the recovery was 100.1 ± 3.7%.

After preliminary experiments (3.4.) and the DoE (3.5.), the results show that the flow rate does not have a significant impact on the release of the API from the diclofenac sodium hydrogel product; however, the volume of the semi-solid adapter does. The impact of the pH is significant on API release, but the effect of the osmolality of the medium is not significant.

After the comprehensive OFAT (see Section 3.4.2 and Section 3.4.3) and DoE (see Section 3.5) investigation of the high-ranked CMPs, the FMEA table was updated according to the previous initial FMEA table (Table 4). On the basis of the updated FMEA table (Table S4), the following CMPs were reclassified from high-ranked to medium or low classes: rate of flow, membrane type, individual flow rate of cells, API%, and the composition of the product.

### 3.7. Investigating Discriminatory Power

**Figure 5:**
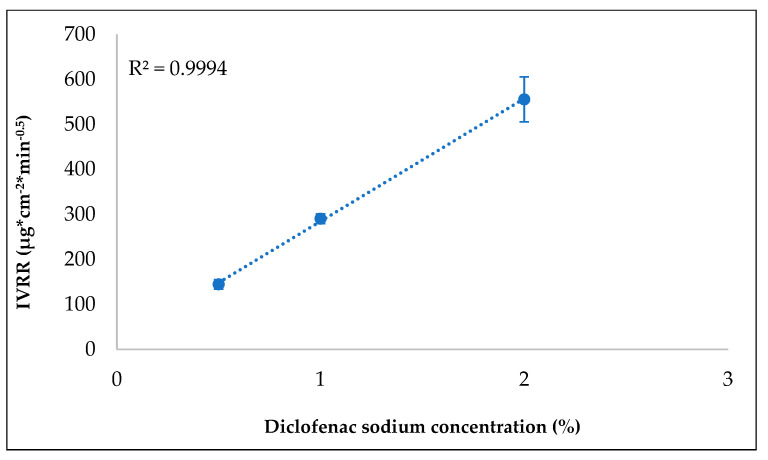
Specificity of the in vitro release test (IVRT).

The discriminatory power was analyzed for the 0.4 mL sample amount. It can be seen that the IVRT method is sensitive because it was capable of detecting different in vitro release rates with respect to the strength of the formulations, and the relationship between the different diclofenac sodium strengths and IVRR is linear (R^2^ = 0.9994) (Figure 5, Figures S5 and S6).

The calculated lower and upper limits fall outside the range of 75–133.33% for both test products; therefore, we confirmed product inequivalence. This is a significant difference between the tests and the reference; therefore, the IVRT method is capable of detecting inequivalence.

## 4. Conclusions

In this study, we showed how the concept of AQbD can be applied in the early stages of IVRT method development in the case of USP apparatus IV. After defining the ATP and selecting CMAs (at least 70% of the active substance applied is released after 6 h, six time points should be obtained in the linear portion of the drug release profile, and the relative standard deviation in the computed released amount of the six vessels was less than or equal to 10%), an initial risk assessment was carried out: with the help of the Ishikawa diagram, more than 100 method parameters were identified that can influence method performance and the quality of the results. FMEA was used to reduce the number of possible parameters down to eight factors: ionic strength, the pH of the medium, membrane type, the rate of flow, sample weight (volume of the SSA), the individual flow rate of cells, API% (0.5, 1, and 2%), and the composition of the product. During the screening process, we examined the impact of these parameters on CMAs independently of each other. These CMPs (the pH of the medium and the sample weight of the product) were given as independent variables in the factorial design. A 2^3^ full factorial design experiment was employed to assess the IVRR and the release efficiency in 6 h.

After the examination, we re-evaluated the risks according to the results and recorded them in the updated FMEA table (Table S4), thus narrowing the method parameters to CMPs. 

On the basis of our results, the amount of the product and the pH were clearly defined as critical parameters during the application of the AQbD approach. At least 70% diclofenac sodium release from the hydrogel (all parallel samples) was achieved within 6 h under all testing conditions; therefore, it meets the ATP requirements. The ATP is capable of satisfying the EMA guideline [1] criteria. On the other hand, the means of operation of USP apparatus IV allows more time points to be applied in order to meet the criterion “6 time points should be obtained in the linear portion of the drug release profile” [1]. Summarizing our results, a robust IVRT test can be developed using the USP apparatus IV, which complies with the international guidelines, but the effect of the pH of the medium and the sample weight on the IVRT results must be analyzed in each case.

## Figures and Tables

**Table 1:** Description of ranking.

Category	Ranking				
	1	2	3	4	5
Occurrence (O)	nearly impossible	randomly occurring	50% chance of occurring	likely to occur	certain to occur
Severity (S)	no effects	insignificant effect	moderate effect	strong effect	severe effect
Detectability (D)	excellent	good	moderate	poor	undetectable

**Table 2:** Analytical target profile of diclofenac sodium topical gel.

ATP Element	Target
Target sample (product name)	Diclofenac sodium 1% topical gel
API name	Diclofenac sodium
Dosage strength	1% (10 mg/g)
Dosage forms	Hydrogel
Route of administration	Topical
Matrix	Propylene glycol (50%), HPMC (1.5%), purified water (47.5%)
Packaging	Plastic tube
Regulatory specification	ICH, EMA (European Medicines Agency), FDA (Food and Drug Administration)
Release/in vitro release test	The release tests should be sensitive to relevant changes in the ingredients and process parameters. They should have adequate release efficiency, release profiles, and reproducibility. They should meet regulatory requirements [1]. Precision RSD ≤ 10% (6 parallel).
Analytical measurements	Analytical measurements: the procedure must be able to accurately quantify diclofenac sodium in IVRT samples over the range of 25–200% of the nominal concentration with an accuracy of 2.0%

**Table 3:** Critical method attributes of diclofenac sodium topical gel.

CMA Parameters	Target	Justification
Release efficiency in 6 h	Q (6 h) ≥ 70%	IVRT is a fundamental tool used to identifyformulation factors that influence the release of the API, an effective method to monitor lot-to-lot changes and stability during development. A draft guideline on the quality and equivalence of topical products described this criterion [1].
Characterize the release profile	6 time points should be obtained in the linear portion of the drug release profile	
RSD% of the released API amount of the 6 parallel samples at given sampling points	RSD ≤ 10% (6 parallel)	RSD values below 10% are considered to be an indication of the good reproducibility of the IVRT method.
Accuracy	Between 98 and 102%	In the case of UHPLC measurements, the weak point of the true value determination is accuracy.
System suitability test of the chromatography system	USP plate count: N ≥ 3000	There is a need for a chromatography system in which the API can properly separate from the matrix components. The plate count has a fundamental impact on the extent of measurement error through the peak’s capability of being integrated. Therefore, the chromatography method should be suitable within the purpose to detect the API in IVRT samples at 25% of the nominal concentration.

**Table 4:** Initial risk assessment for in vitro release test (IVRT) method development (high risk). F probability of occurrence of the excursion = 1 (low), 5 (high); S severity of excursion = 1 (low), 5 (high); D detection of excursion = 1 (easy), 5 (hard); RPN risk priority number = F × S × D.

Method Parameter	Critical Method Attributes	Cause of the Deviation	Effect of the Deviation	F (Occurrence)	S (Severity)	D (Perceptibility)	RPN	Action/Strategy of Risk Decrease
Release test								
Ionic strength of the medium	min. 70% (Q)—6 h	The gelling agent is HPMC	Release might change	4	5	4	80	We need to investigate the effect of the ionic strength of the medium (pH 7.4 PBS ± NaCl).
Ionic strength of the medium	6 time points should be obtained in the linear portion of the drug release profile	The gelling agent is HPMC	Release might change	4	5	4	80	We need to investigate the effect of the ionic strength of the medium (pH 7.4 PBS ± NaCl).
pH of the medium	min. 70% (Q)—6 h	Changing the pH of the medium	RSD might be increasing; outliers below 70%	3	5	4	60	Controlled parameter: prescription is needed to make the medium pH 7.4 ± 0.5. Investigation of the effect of pH change is needed.
Membrane type	min. 70% (Q)—6 h	Different membrane and manufacturer	The membrane should be inert and not be rate-limiting to active substance release	4	5	3	60	We need to investigate the inertness of the membrane in pH 7.4 PBS medium.
Rate of flow	min. 70% (Q)—6 h	The increase in the rate of flow, maintaining the concentration gradient, results in faster drug release	Release kinetic might change; increase or decrease in RSD	5	5	3	75	We need to investigate the effect of the flow rate changing (4 mL/min to 8 mL/min).
Rate of flow	6 time points should be obtained in the linear portion of the drug release profile	Quicker flowing causes quicker release	Release kinetic might change	5	5	3	75	We need to investigate the effect of the flow rate changing (4 mL/min to 8 mL/ min).
Sample weight (0.4 mL or 1.2 mL SSA)	min. 70% (Q)—6 h	Different size of SSA	Sample weight increasing, leading to release kinetic change/release rate change	5	5	3	75	We need to investigate the effect of the sample weight (0.4 mL or 1.2 mL SSA).
Sample weight (0.4 mL or 1.2 mL SSA)	6 time points should be obtained in the linear portion of the drug release profile	Different size of SSA	Sample weight increasingleading to release kinetic change/release rate change	5	5	3	75	We need to investigate the effect of the sample weight (0.4 mL or 1.2 mL SSA).
Individual flow rate of cells	min. 70% (Q)—6 h	The release of API might be changing cell by cell	RSD might be increasing; outliers above 70%	3	5	5	75	Measuring the flow rate cell by cell of the release and calculating the release with the measured flow rate. Conducting training about how to assemble the cells. Annual maintenance.
Individual flow rate of cells	6 time points should be obtained in the linear portion of the drug release profile	The release of API might be changing cell by cell	RSD might be increasing; fluctuating release curve is caused by RSD%	3	5	5	75	Measuring the flow rate cell by cell of the dissolution and calculating the dissolution with the measured flow rate.
Individual flow rate of cells	RSDConc ≤ 10% (6 vessels)	The release of API might be changing cell by cell	Fluctuating release curve is caused by RSD%	3	5	5	75	Conducting training about how to assemble the cells. Annual maintenance.
API%	min. 70% (Q)—6 h	Sink conditions must be ensured in the receptor medium	Limited drug solubility effects can play a major role in the control of API release	5	5	3	75	What is the hydrogel diclofenac sodium’s maximum dosage that we are going to use?
API%	6 time points should be obtained in the linear portion of the drug release profile	The method’s requirement is to detect different IVRRs according to the strength of the formulations	The IVRT method might not be sensitive	4	5	3	60	We need to investigate the discriminatory ability of the IVRT method (different formulation strengths: 0.5, 1, and 2%).
Composition of the product	min. 70% (Q)—6 h	Gelling agent type	Release might change	4	5	3	60	We need to prescribe that the matrix is fixed.

**Table 5:** In vitro release test (IVRT) results of preliminary experiments.

Media	Osmolality	Flow Rate	Semi-Solid Adapter	Computed Released Amount at the End of the Experiment at 6 h			IVRR			Lag Time		
				Mean	SD	RSD	Mean	SD	RSD	Mean	SD	RSD
	mOsmol/kg	mL/min	mL	%	%	%	µg × cm^−2^ × min^0.5^	µg × cm^−2^ × min^0.5^	%	min	min	%
pH 7.4 PBS	279.5	4	1.2	75.5	3.5	4.6	420.2	21.6	5.2	22.9	1.3	5.5
pH 7.4 PBS	279.5	8	0.4	100.6	3.6	3.6	273.8	10.2	3.7	8.6	1.2	14.0
pH 7.4 PBS	279.5	4	0.4	99.5	4.6	4.7	278.5	10.5	3.8	11.7	0.7	6.0
pH 7.4 PBS	279.5	8	1.2	81.2	3.5	4.3	446.7	18.2	4.1	20.1	1.5	7.3
pH 7.4 PBS + NaCl	769.3	8	0.4	94.4	2.2	2.3	274.6	9.5	3.5	9.7	0.6	6.3
pH 7.4 PBS–NaCl	99.3	8	0.4	91.3	1.8	1.9	275.6	4.5	1.6	8.3	0.5	5.7
pH 6.9 PBS	274.5	8	0.4	86.5	2.5	2.9	262.1	8.6	3.3	9.4	0.8	8.2
pH 7.9 PBS	277.0	8	0.4	99.5	3.0	3.0	299.1	8.9	3.0	9.7	0.7	7.0

**Table 6:** Experimental design matrix according to a 2^3^ full factorial design.

Experiment	Flow Rate (mL/min)	Volume of SSA (mL)	pH
1	4.00	0.40	7.40
2	8.00	0.40	7.40
3	4.00	1.20	7.40
4	8.00	1.20	7.40
5	4.00	0.40	7.90
6	8.00	0.40	7.90
7	4.00	1.20	7.90
8	8.00	1.20	7.90

**Table 7:** Results of the statistical analysis for in vitro release rate (IVRR) (µg × cm^−2^ × min^−0.5^).

Factor	Effect	t(32)	*p*	Coefficient	Standard Error Coefficient
Mean/intercept	365.9818	137.8254	0.0000	365.9818	2.6554
(1) A: Flow rate (mL/min)	4.2335	0.7971	0.4312	2.1168	2.6554
(2) B: Volume of SSA (mL)	158.0885	29.7673	0.0000	79.0443	2.6554
(3) C: pH	23.9005	4.5004	0.0001	11.9503	2.6554
1 by 2	6.8665	1.2929	0.2053	3.4333	2.6554
1 by 3	−3.5875	−0.6755	0.5042	−1.7938	2.6554
2 by 3	0.5395	0.1016	0.9197	0.2698	2.6554
1 × 2 × 3	−7.2765	−1.3701	0.1802	−3.6383	2.6554

**Table 8:** Results of the statistical analysis for release efficiency in 6 h (%).

Factor	Effect	t(32)	*p*	Coefficient	Standard Error Coefficient
Mean/intercept	88.2920	150.1317	0.0000	88.2920	0.5881
(1) A: Flow rate (mL/min)	1.8480	1.5712	0.1260	0.9240	0.5881
(2) B: Volume of SSA (mL)	−22.4020	−19.0462	0.0000	−11.2010	0.5881
(3) C: pH	−1.5510	−1.3187	0.1966	−0.7755	0.5881
1 by 2	0.9680	0.8230	0.4166	0.4840	0.5881
1 by 3	−1.3130	−1.1163	0.2726	−0.6565	0.5881
2 by 3	−0.9070	−0.7711	0.4463	−0.4535	0.5881
1 × 2 × 3	−1.1150	−0.9480	0.3502	−0.5575	0.5881

## Data Availability

The data presented in this study are available on request from the corresponding author.

## Supplementary Materials

The following supporting information can be downloaded at: https://www.mdpi.com/article/10.3390/pharmaceutics14040707/s1, Figure S1: Standard Pareto chart showing the effects of independent variables on in vitro release rate (IVRR) (µg × cm^−2^ × min^−0.5^); Figure S2: Response surface (3D) plot (X2-X3-Y1) of the effects of variables on in vitro release rate (IVRR) (µg × cm^−2^ × min^−0.5^); Figure S3: Standard Pareto chart showing the effects of independent variables on release efficiency in 6 h (%); Figure S4: Response surface (3D) plot (X1-X2-Y1) of the effects of variables on release efficiency in 6 h (%); Figure S5: Response surface (3D) plot (X1-X3-Y1) of the effects of variables on release efficiency in 6 h (%); Figure S6: Response surface (3D) plot (X1-X2-Y1) of the effects of variables on in vitro release rate (IVRR) (µg × cm^−2^ × min^−0.5^); Figure S7: Response surface (3D) plot (X1-X3-Y1) of the effects of variables on in vitro release rate (IVRR) (µg × cm^−2^ × min^−0.5^); Figure S8: Response surface (3D) plot (X2-X3-Y1) of the effects of variables on release efficiency in 6 h (%); Table S1: Confirmation of the sink condition; Table S2: Osmolality of the applied media in the in vitro release test (IVRT) studies; Table S3: Effect of the pH for in vitro release rate (IVRR). Statistical parameters—one-way analysis of variance test; Table S4: Updated failure modes and effects analysis (FMEA) table after the investigation of CMPs; Table S5: Sensitivity of the in vitro release test (IVRT) method: in vitro release rate (IVRR) of the diclofenac sodium 0.5, 1, and 2% hydrogel measured with USP apparatus IV; Table S6: The discriminatory power of the in vitro release test (IVRT) method calculated the upper and the lower limits of the 90% confidence interval.
