# Supplementary material for: Analytical Quality by Design (AQbD) Approach to the Development of In Vitro Release Test for Topical Hydrogel
Source: Pharmaceutics. 2022 Mar 26;14(4):707. doi: 10.3390/pharmaceutics14040707 (PMC9026520; doi:10.3390/pharmaceutics14040707)
Supplement: Supplementary file 1 [file pharmaceutics-14-00707-s001.zip › pharmaceutics-1632872-supplementary.pdf]

pharmaceutics

Supplementary Materials: Analytical quality by design (AQbD) approach to the development of in vitro release test for topical hydrogel

Réka Szoleczky, Mária Budai-Szűcs, Erzsébet Csányi, Szilvia Berkó, Péter Tonka-Nagy, Ildikó Csóka and Anita Kovács*

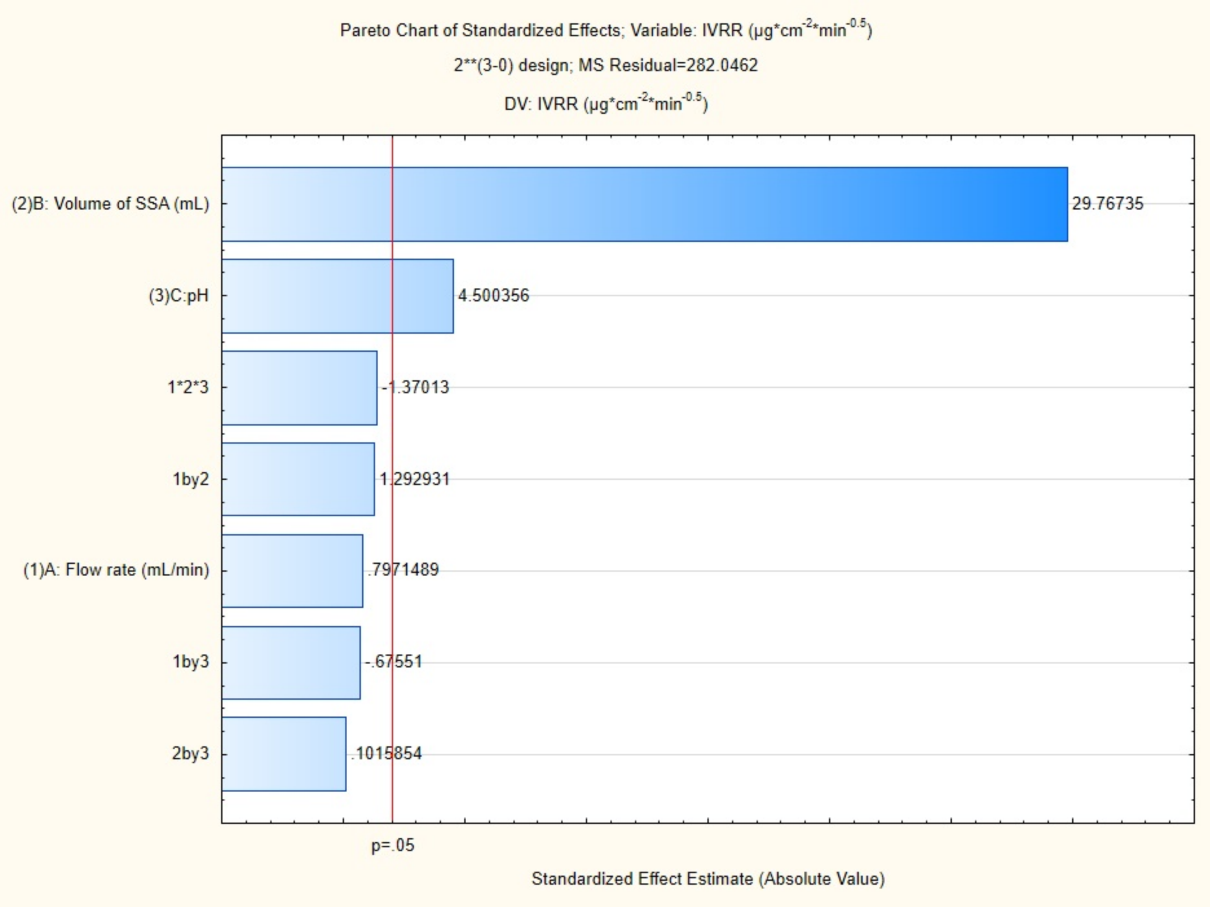

Figure S1. Standard Pareto chart showing the effects of independent variables on in vitro release rate (IVRR) (µg*cm^{-2}*min$^{-0.5}$).

pharmaceutics

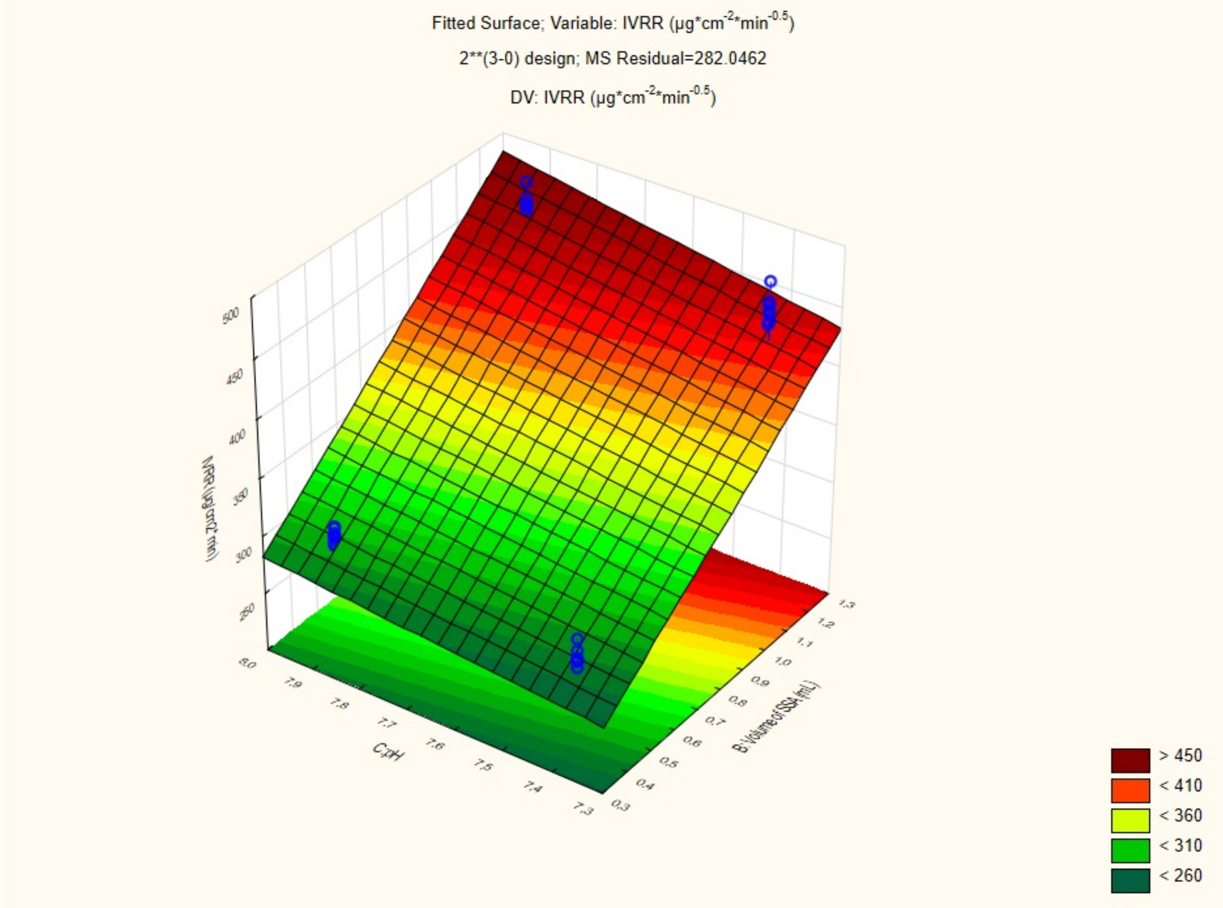

Figure S2. Response surface (3D) plot (X2-X3-Y1) of the effects of variables on in vitro release rate (IVRR) (µg*cm^{-2}*min$^{-0.5}$).

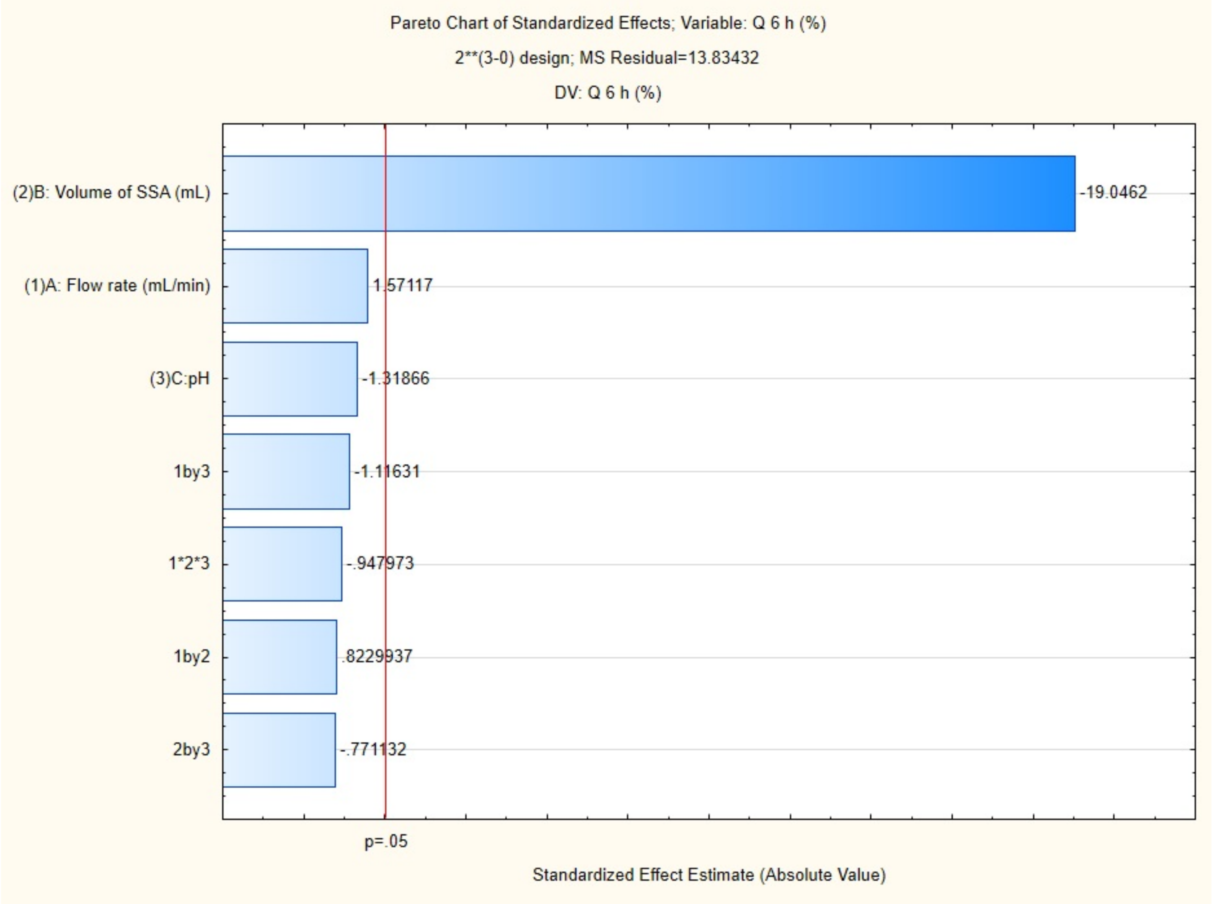

Figure S3. Standard Pareto chart showing the effects of independent variables on release efficiency in 6 hours (%).

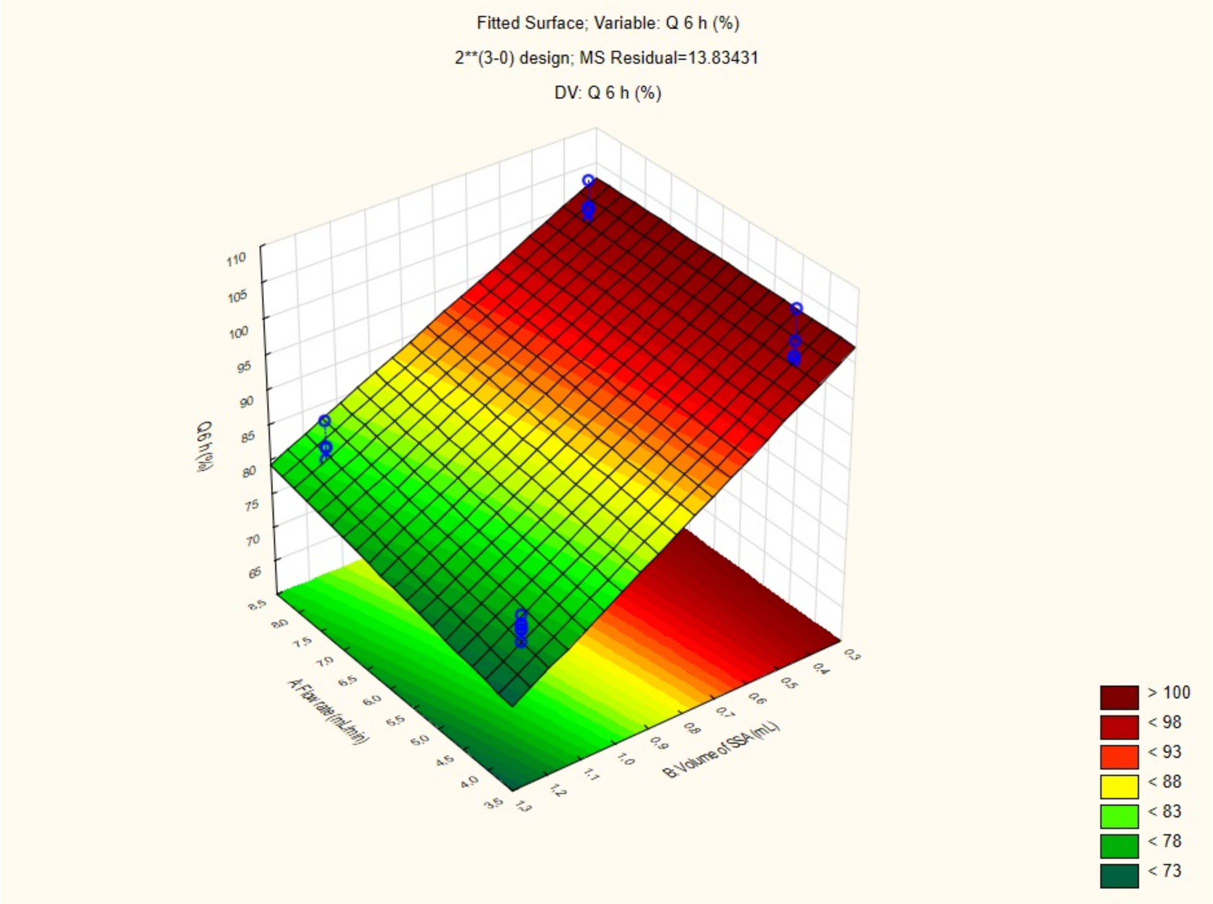

Figure S4. Response surface (3D) plot (X1-X2-Y1) of the effects of variables on release efficiency in 6 hours (%).

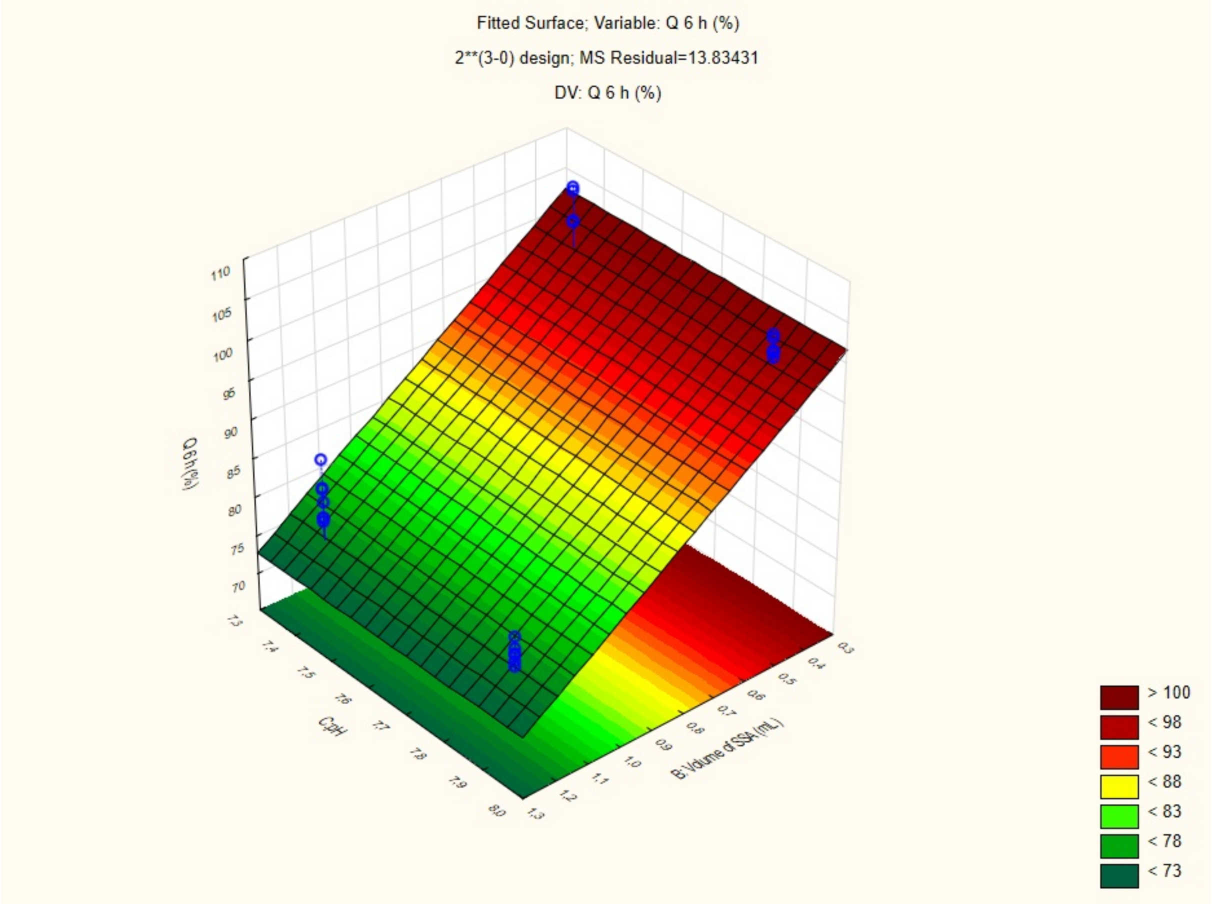

Figure S5. Response surface (3D) plot (X1-X3-Y1) of the effects of variables on release efficiency in 6 h (%).

pharmaceutics

MDPI

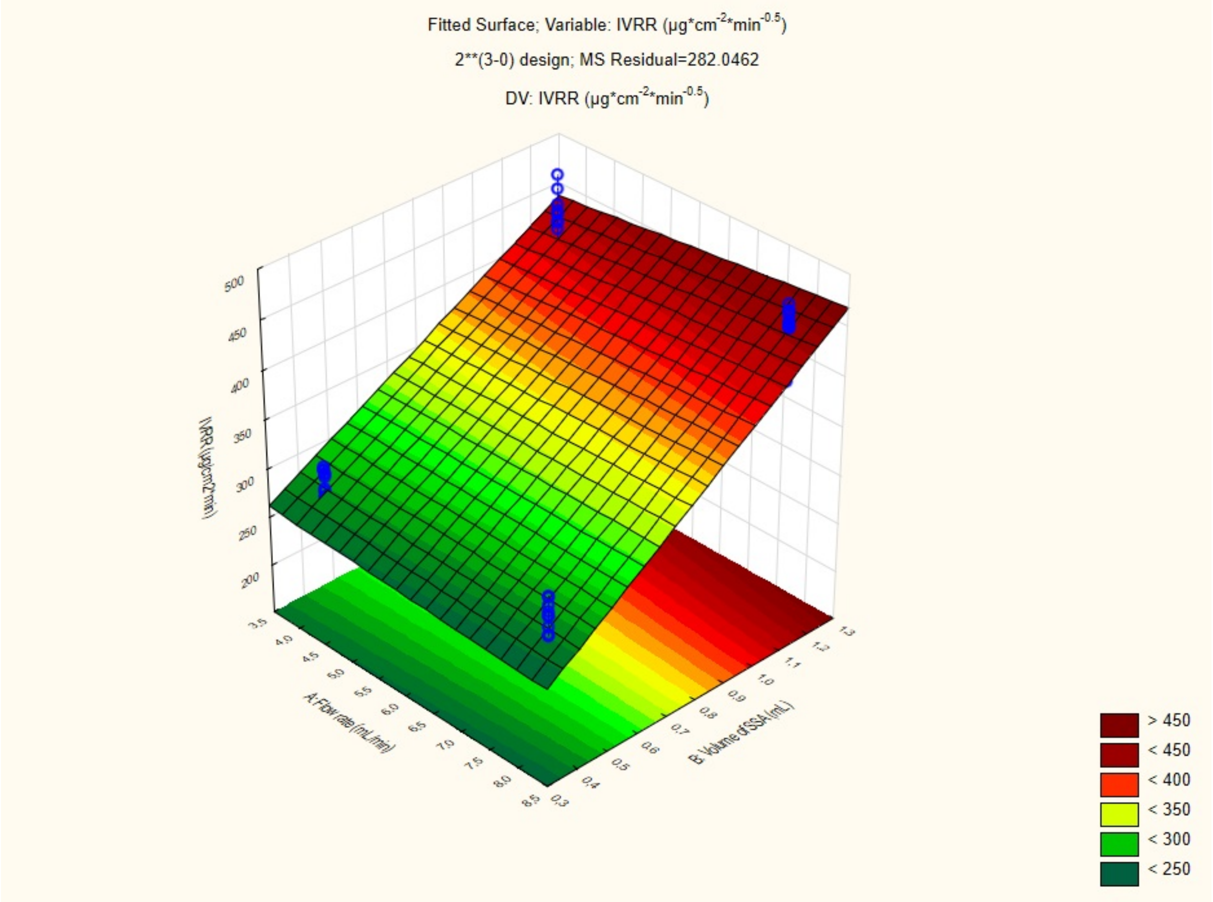

Figure S6. Response surface (3D) plot (X1-X2-Y1) of the effects of variables on in vitro release rate (IVRR) (µg*cm^{-2}*min$^{-0.5}$).

pharmaceutics

MDPI

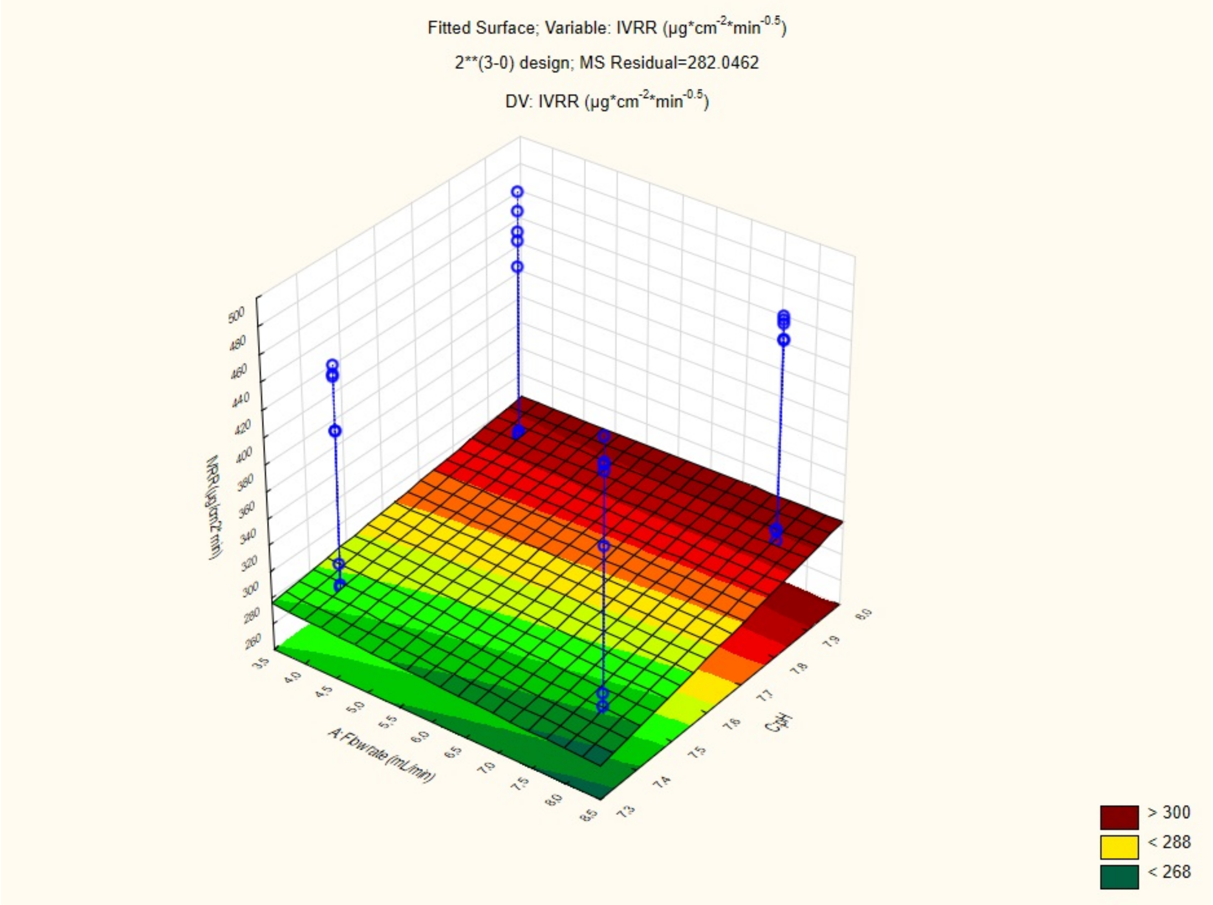

Figure S7. Response surface (3D) plot (X1-X3-Y1) of the effects of variables on in vitro release rate (IVRR) ($\mu g{*}cm^{-2}{*}min^{-0.5}$).

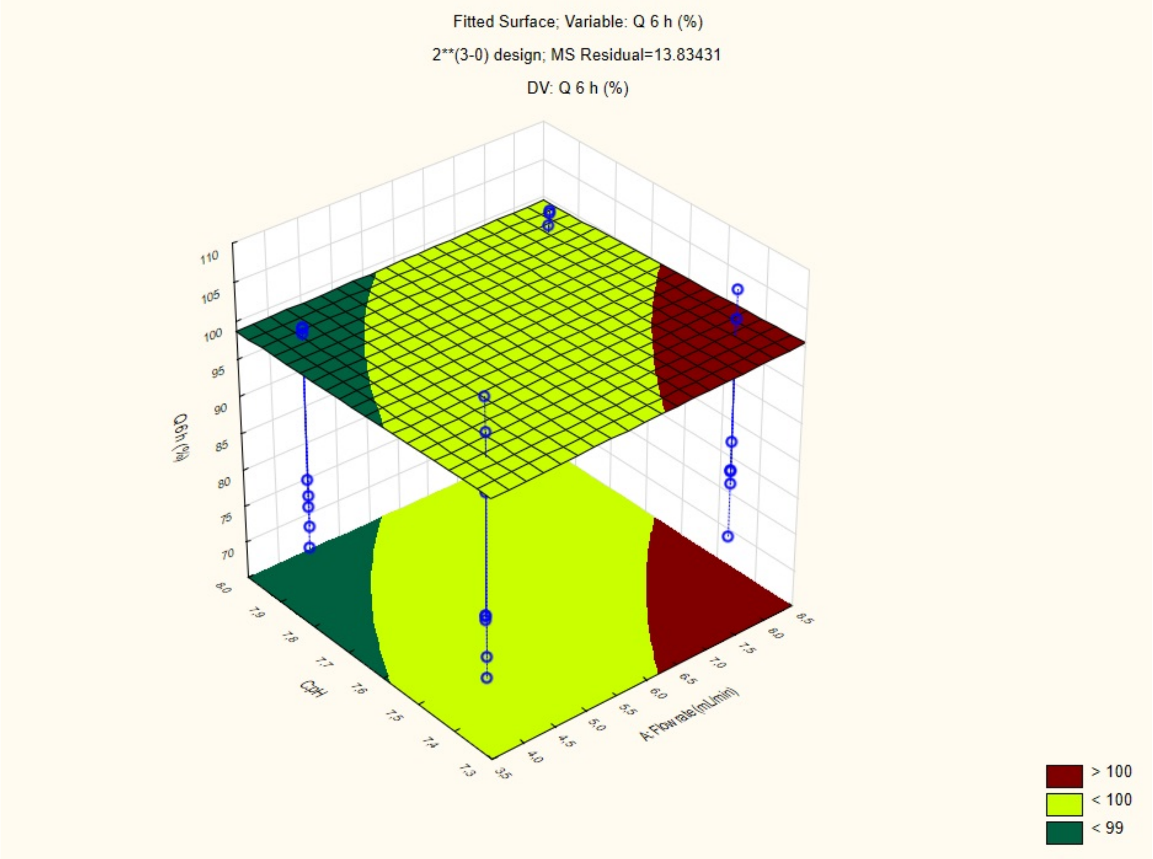

Figure S8. Response surface (3D) plot (X2-X3-Y1) of the effects of variables on release efficiency in 6 h (%).

Table S1. Confirmation of the sink condition.

Parameters	USP Apparatus IV
Maximum dosage strength (%)	2
Volume of the cell (mL)	10.2
Maximum sample weight (mg)	1300
Maximum concentration of the API in the receptor medium (mg/mL)	2.6
Solubility of API in pH 7.4 PBS mg/mL	8.2 ± 0.7*
Solubility of API in pH 7.4 PBS divided maximum concentration of the API in the receptor medium (-)	3.2

*M. Miranda, A. A. C. C. Pais, C. Cardoso, C. Vitorino, aQbD as a platform for IVRT method development–A regulatory oriented approach. International Journal of Pharmaceutics. 572, (2019) 118695. https://doi.org/10.1016/j.ijpharm.2019.118695.

Table S2. Osmolality of the applied media in the in vitro release test (IVRT) studies.

Name of the Medium	Osmolality of the Medium Mean (mOsmol/kg)
pH 7.4 PBS	279.5
pH 6.9 PBS	274.5
pH 7.9 PBS	277.0
pH 7.4 PBS + NaCl	769.3
pH 7.4 PBS - NaCl	99.3

pharmaceutics

MDPI

Table S3. Effect of the pH for in vitro release rate (IVRR). Statistical parameters – one-way analysis of variance test.

Effect	Effect (F/R)	Sum of Squares	Degr. Of Freedom	Mean Square	Den. Syn. Error df	Den. Syn. Error MS	F	p
Intercept	Fixed	1230515	1	1230515	2,00067	1796,229	685,0547	0,001454
pH	Random	3604	2	1802	13,00000	87,250	20,6551	0,000092
Error		1134	13	87	-	-	-	-

Table S4. Updated failure mode effects analysis (FMEA) table after the investigation of CMPs. F probability of occurrence of the excursion = 1 (low), 5 (high); S severity of excursion = 1 (low), 5 (high); D detection of excursion = 1 (easy), 5 (hard); RPN risk priority number = F×S×D; 1–29 low risk (green), 30–59 medium risk (yellow), 60–125 high risk (orange).

Method parameter	Critical Method attributes	Cause Ofthe Deviation	Effect of the Deviation	F (occurrence)	S (severity)	D (perceptibility)	RPN	Action/Strategy of Risk Decrease
**Release test**								
Ionic strength of the medium	min. 70% (Q)-6 h	The gelling agent is HPMC	Release might change	4	2	4	32	According to OFAT studies (see Section 3.4.3).
Ionic strength of the medium	6 time points should be obtained in the linear portion of the drug release profile	The gelling agent is HPMC	Release might change	4	1	4	16	According to OFAT studies (see Section 3.4.3).
pH of the medium	min. 70% (Q)-6 h	Changing the pH of the medium	RSD might be increasing. Outliers above 70%.	3	5	4	60	According to OFAT studies (see Section 3.4.3) and DoE (see Section 3.5.)
Membrane type	min. 70% (Q)-6 h	Different membrane and manufacturer	The membrane should be inert and not be rate-limiting to active	5	1	3	15	According to membrane inertness study, see Section 3.4.1.

			substance release				
Rate of flow	min. 70% (Q)-6 h	The increase in the rate of flow, maintaining concentration gradient, results in faster drug release	Release kinetic might change. Increase or decrease of RSD	5	1	3	15	According to OFAT studies (see Section 3.4.2.) and DoE (see Section 3.5.)
Rate of flow	6 time points should be obtained in the linear portion of the drug release profile	Quicker flowing causes quicker release	Release kinetic might change	5	1	3	15	According to OFAT studies (see Section 3.4.2.) and DoE (see Section 3.5.)
Sample weight (0.4 mL or 1.2 mL SSA)	min. 70% (Q)-6 h	Different size of SSA	Sample weight increasing, therefore release kinetic change / release rate change	5	5	3	75	According to OFAT studies (see Section 3.4.2.) and DoE (see Section 3.5.)
Sample weight (0.4 mL or 1.2 mL SSA)	6 time points should be obtained in the linear portion of the drug release profile	Different size of SSA	Sample weight increasing, therefore release kinetic change / release rate change	5	5	3	75	According to OFAT studies (see Section 3.4.2.) and DoE (see Section 3.5.)

Individual flow rate of cells	min. 70% (Q)-6 h	The release of API might be changing cell by cell	RSD might be increasing. Outliers above 70%.	3	3	5	45	Controlled parameter: it was prescribed to measure the flow rate cell by cell of the release and calculate the release with the measured flow rate.
Individual flow rate of cells	6 time points should be obtained in the linear portion of the drug release profile	The release of API might be changing cell by cell	RSD might be increasing. Fluctuating release curve is caused by RSD %	3	3	5	45	Controlled parameter: it was prescribed to measure the flow rate cell by cell of the release and calculate the release with the measured flow rate.
Individual flow rate of cells	RSDConc ≤ 10 % (6 vessels)	The release of API might be changing cell by cell	Fluctuating release curve is caused by RSD %	3	3	5	45	Controlled parameter: it was prescribed to measure the flow rate cell by cell of the release and calculate the release with the measured flow rate.

API %	min. 70% (Q)-6 h	Sink conditions have to be ensured in the receptor medium	Limited drug solubility effects can play a major role in the control of API release	5	1	3	15	According to discriminatory power studies, see Section 3.7. The LOQ was measured (0.12 µg/mL) and adequate for ATP.
API %	6 time points should be obtained in the linear portion of the drug release profile	The method's requirement is to detect different IVRRs according to the strength of the formulations.	The IVRT method might not be sensitive.	4	1	3	12	According to discriminatory power studies, see Section 3.7.
Composition of the product	min. 70% (Q)-6 h	Gelling agent type	Release might change	4	3	3	36	Fixed matrix was prescribed.

pharmaceutics

MDPI

Table S5. Sensitivity of the in vitro release test (IVRT) method: in vitro release rate (IVRR) of the diclofenac sodium 0.5, 1 and 2 % hydrogel measured with USP apparatus IV.

Diclofenac Sodium Concentration %	IVRR µg*cm^{-2}*min$^{-0,5}$	Mean of the IVRR µg*cm^{-2}*min$^{-0,5}$	SD of the IVRR µg*cm^{-2}*min$^{-0,5}$	RSD of the IVRR %
0.5	137.34<br>160.91<br>138.12<br>152.88<br>133.55<br>144.43	144.54	10.50	7.26
1	309.39<br>289.96<br>292.30<br>284.38<br>276.42<br>290.86	290.55	10.92	3.76
2	573.25<br>595.43<br>477.18<br>602.49<br>511.19<br>574.37	555.65	50.14	9.02

Table S6. The discriminatory power of the in vitro release test (IVRT) method calculated the upper and the lower limits of the 90% confidence interval.

Diclofenac Sodium Concentration (%) of the Test / Reference Product	90 CI%		Decision
	Lower limit	Upper limit	
0.5/1 %	47.0	53.8	inequivalence
2/1 %	174.9	207.4	inequivalence
